# Septic Shock in Advanced Age: Transcriptome Analysis Reveals Altered Molecular Signatures in Neutrophil Granulocytes

**DOI:** 10.1371/journal.pone.0128341

**Published:** 2015-06-05

**Authors:** Diogo Vieira da Silva Pellegrina, Patricia Severino, Hermes Vieira Barbeiro, Flávia Maziero Andreghetto, Irineu Tadeu Velasco, Heraldo Possolo de Souza, Marcel Cerqueira César Machado, Eduardo Moraes Reis, Fabiano Pinheiro da Silva

**Affiliations:** 1 Programa Interunidades de Pós-Graduação em Bioinformática, Universidade de São Paulo, São Paulo, Brazil; 2 Instituto Israelita de Ensino e Pesquisa, Hospital Israelita Albert Einstein, São Paulo, Brazil; 3 Departamento de Emergências Clínicas, Faculdade de Medicina da Universidade de São Paulo, São Paulo, Brazil; 4 Departamento de Bioquímica, Instituto de Química, Universidade de São Paulo, São Paulo, Brazil; University of Leicester, UNITED KINGDOM

## Abstract

Sepsis is one of the highest causes of mortality in hospitalized people and a common complication in both surgical and clinical patients admitted to hospital for non-infectious reasons. Sepsis is especially common in older people and its incidence is likely to increase substantially as a population ages. Despite its increased prevalence and mortality in older people, immune responses in the elderly during septic shock appear similar to that in younger patients. The purpose of this study was to conduct a genome-wide gene expression analysis of circulating neutrophils from old and young septic patients to better understand how aged individuals respond to severe infectious insult. We detected several genes whose expression could be used to differentiate immune responses of the elderly from those of young people, including genes related to oxidative phosphorylation, mitochondrial dysfunction and TGF-β signaling, among others. Our results identify major molecular pathways that are particularly affected in the elderly during sepsis, which might have a pivotal role in worsening clinical outcomes compared with young people with sepsis.

## Introduction

Sepsis is a complex syndrome triggered by infection and characterized by the massive deregulation of immunological networks [1]. Septic patients have a mean age of approximately 65 years [2] and the incidence of sepsis and its risk of mortality increase significantly with advanced age [3, 4]. Factors that might contribute to the increased risk and incidence in the elderly include defects in the integrity of epithelial barriers, decreased gag and cough reflexes, altered levels of consciousness, immobility, concomitant medical diseases, a dependency on invasive medical devices, diminished physiological reserves, endocrine deficiencies, and malnutrition [5, 6].

Age-related defects in immunity are caused by major defects in cell-mediated and humoral effector functions [7]. Aging causes changes in adaptive immunity associated with a shift of the T cell repertoire from a naive phenotype to memory T cells [3] and from type 1 to type 2 responses [8, 9]. Numbers of B and plasma cells gradually decrease with age, while immunoglobulin levels increase [10].

Innate immunity was previously considered to be well-preserved in the elderly [11], but recent studies have also demonstrated significant alterations in these components [3]. Studies have also indicated the altered expression and function of Toll-like receptors (TLRs) caused by aging, altering the host’s response to pathogens [12]. Elevated levels of basal inflammation [13, 14], defective activation of mitogen-activated protein kinases (MAPKs) [15], increased number of apoptotic cells [16], defects in phagocytosis, generation of reactive oxygen species (ROS) and impaired costimulatory molecule expression have also been reported in the elderly [17]. Indeed, recent studies have indicated that older adults have elevated levels of pro-inflammatory cytokines, clotting factors and acute phase reactants in the steady state [18–20].

The inflammatory response of the elderly following infection, however, remains under debate. Animal studies demonstrated that mortality, inflammation, hypothermia, apoptosis and disseminated intravascular coagulation are increased in aged animals subjected to experimental models of sepsis [21]. It is intriguing that despite the well characterized aspects of immunosenescence and the exaggerated inflammatory response detected in septic aged rodents [22, 23], clinical studies conducted in humans (including those from our group) observed a similar immune profile when old and young septic critically ill patients were compared [24–27].

The purpose of this study was to perform a genome-wide gene expression analysis of neutrophils obtained from elderly and young patients in septic shock, to investigate the potential differences in cell activation that might explain the altered status of immune and inflammatory systems in advanced aged patients with sepsis. We decided to focus on neutrophils because recent reports suggested their function was particularly altered in the elderly [28].

## Patients and Methods

### Study design

The current study was a prospective cohort study, conducted in the Hospital das Clínicas Intensive Care Units (University of Sao Paulo, Brazil). Blood samples were obtained from six aged septic patients (age range 65–78 years old), six young septic patients (age range 22–35 years old), six healthy aged volunteers (age range 60–82 years old) and six healthy younger individuals (age range 20–35 years old). Patients’ profiles are described in the S1 Table. All cases of sepsis were in patients with a clinical illness and did not include patients admitted for trauma or surgical reasons. Reasons for the majority of admissions included in this study were sepsis, stroke, altered levels of consciousness, pulmonary edema, and asthma and/or chronic obstructive pulmonary disease. Patients who were less than 18 years old, pregnant, HIV-positive, or in end-of-life conditions were excluded. Patients with disseminated malignancies or advanced hepatic disease, those receiving chemotherapy and those who refused to participate in this study were also excluded. Septic shock was defined according to the criteria of the American College of Chest Physicians/Society of Critical Care Medicine (ACCP/SCCM) Consensus Conference Committee proposed in 1992 [29].

The study protocol was approved by the Hospital das Clínicas Ethics Committee. Patients (or their close relatives) received detailed explanations and provided written consent prior to inclusion in the study (Protocol # 1207/09).

### RNA extraction and microarray experiments

All blood samples were sent to our laboratory for processing immediately after collection. The anticoagulant-treated blood was layered on the Ficoll-Paque PLUS solution (GE Healthcare) and centrifuged for a short period of time. Differential migration during centrifugation results in the formation of layers containing different cell types. The bottom layer contains erythrocytes that have been aggregated by the Ficoll and, therefore, sediment completely through the Ficoll-Paque PLUS. The layer immediately above the erythrocyte layer contains the granulocytes, which at the osmotic pressure of the Ficoll-Paque PLUS solution attain a density great enough to migrate through the Ficoll-Paque PLUS layer.

After Ficoll-Paque PLUS (GE Healthcare) density gradient centrifugation, we separated the second layer containing the granulocytes. This layer was transferred to new tubes, diluted in lysis buffer and kept on ice for 10 minutes. After centrifugation at 290 x *g* for 10 minutes at 4°C the pellet was resuspended in lysis buffer and kept on ice for additional 10 minutes. A new centrifugation step was performed at 2500 x *g* for 2 minutes at room temperature and the samples were washed with phosphate-buffered saline (PBS). Finally, the samples were centrifuged at 1500 x *g* for 2 minutes at room temperature and the pellet was resuspended in Trizol (Life Technology, Carlsbad, USA) and stored at -80°C.

Total RNA was isolated following the manufacturer’s protocol and its integrity and concentration were assessed using the Agilent 2100 Bioanalyzer and the RNA 6000 Nano Kit (Agilent Technologies, Santa Clara, CA, USA). Expression levels of protein-coding genes and long noncoding RNAs (lncRNA) were evaluated using the SurePrint G3 Human Gene Expression 8x60K v2 Microarray (design ID # 039494) and the Low Input Quick Amp Labeling kit, following a two-color labeling protocol (Agilent Technologies). Cyanine-3 labeled RNA from each sample and cyanine-5 labeled reference RNA (Universal Human Reference RNA, Agilent, cat #740000) were combined and hybridized to the microarrays following the manufacturer’s protocols.

### Data processing and filtering

Microarrays were scanned using the SureScan Microarray Scanner (Agilent Technologies) and images were processed using the Feature Extraction Software v12 (Agilent Technologies) for quality control, determination of feature intensities and ratios, and for background correction. The LOWESS procedure was used for data normalization and transformation [30]. We only considered features that were considered “well above background” in at least five (of six) subjects in at least one study group (young adults with sepsis, control young adults, elderly with sepsis, and control elderly). After data filtering and processing, 16,698 probes remained. A Pearson correlation between those filtered features was calculated for each pair of subjects. Overall, we observed a good correlation between any pair of samples (minimum r = 0.88) and a higher correlation between samples from the same group (mean intragroup correlation = 0.96) compared with different groups of samples (mean intergroup correlation = 0.93). Moreover, the samples grouped by disease status and age using unsupervised hierarchical clustering (S1 Fig) further indicating the good quality of the data.

A number of probes (8192) could not be assigned to the known or predicted lncRNA sequences annotated in public databases (Genbank RefSeq, Emsembl). These poorly annotated probes were categorized as “unannotated” and were not analyzed further. The raw and processed microarray datasets are deposited at the Gene Expression Omnibus (accession number GSE67652 associated to platform GPL16699).

### Statistical analysis

We performed two sets of differential gene expression analyses. One analysis was to identify genes deregulated in young and elderly sepsis subjects compared with matched healthy controls. A second analysis searched for genes deregulated in septic or healthy elderly subjects compared with matched young controls. In each analysis, a gene was considered differentially expressed when it was identified by a combination of two statistical methods, namely Significance Analysis of Microarrays [31] and rank product [32], using publicly available R packages [33]. To limit the number of false-positives we only considered for further analysis genes identified as differentially expressed with a *P-*value of *p* ≤ 0.01 by both methods.

### Functional annotation and pathway analysis

Lists of differentially expressed genes were annotated and investigated to identify the enrichment of particular gene categories or pathways using the Ingenuity Pathway Analysis suite (IPA, Qiagen, Redwood City, www.qiagen.com/ingenuity). IPA Upstream Analysis was used to identify putative upstream regulators and to predict whether they were activated or inhibited, given the observed gene expression changes in the experimental dataset, and the expected causal effects, which were compiled from the literature in Ingenuity’s Knowledge Base.

### Relative Quantification of gene expression levels using Real Time-PCR

To validate the microarray expression data, selected genes were subjected to quantitative Real Time-PCR using the TaqMan assay system (Life Technologies). Briefly, 100 ng of total RNA was subjected to reverse transcription using the High-Capacity cDNA Reverse Transcription Kit (Life Technologies) following the manufacturer’s protocol. Specific TaqMan Gene Expression Assays (Life Technologies) were used for detecting gene expression levels of TGFB1 (Hs00998133), SRC (Hs1082246), HDAC4 (Hs01041638), CREBBP (Hs00231733), NDUFA4 (Hs00800172), INHBB (Hs00173582), BMP7 (Hs00233476) and SDHC (Hs01698067). PCR was carried out following the protocol recommended for the TaqMan Universal PCR Master Mix (Life Technologies) in an AB7500 thermocycler (Life Technologies). For each sample, the expression data of each candidate gene was normalized to 18S rRNA expression (Eukaryotic 18S rRNA Endogenous Control, Life Technologies). For relative quantification we used the comparative ∆Ct method [34]. The Student’s *t*-test (*p*-value < 0.05) was used to determine the statistical significance of expression differences between the sample groups.

## Results

The microarray in this study contained 58,717 probes that included protein-coding mRNAs (36,075), long noncoding RNAs (14,450), and a number of poorly annotated transcripts (8192) (Table 1). Approximately 28.4% of all probes were detected in at least one group of samples. A smaller fraction of lncRNAs (10%) was detected compared with protein-coding mRNAs (82%) (Table 1), which is in line with the more tissue-specific and less abundant nature of these transcripts [35, 36].

**Table 1:** Expressed probes in the array according to gene type and sample group.

Probe type	Number of probes in the array	Number of detected probes*	Control vs. Sepsis**	Control vs. Sepsis**	Elderly vs. Young Adults**	Elderly vs. Young Adults**
			Elderly	Young	Sepsis	Control
Protein-coding mRNA	36,075	13,622	1,407	1,631	375	277
Long noncoding RNA	14,450	1,604	165	155	33	112
Unannotated***	8,192	1,472	230	188	13	87
Total	58,717	16,698	1,802	1,974	421	476

*To be considered “detected” a probe was measured with the ‘Well Above Background’ flag in at least five of six samples in at least one group.

** Number of differentially expressed probes in each group: To be considered “differentially expressed” a probe should have a *p*-value ≤ 0.01 assigned by two different methods (SAM and RankProduct).

***Probes were labeled as “unannotated” when not clearly categorized as coding or non-coding in the microarray annotation table.

**Fig 1:**
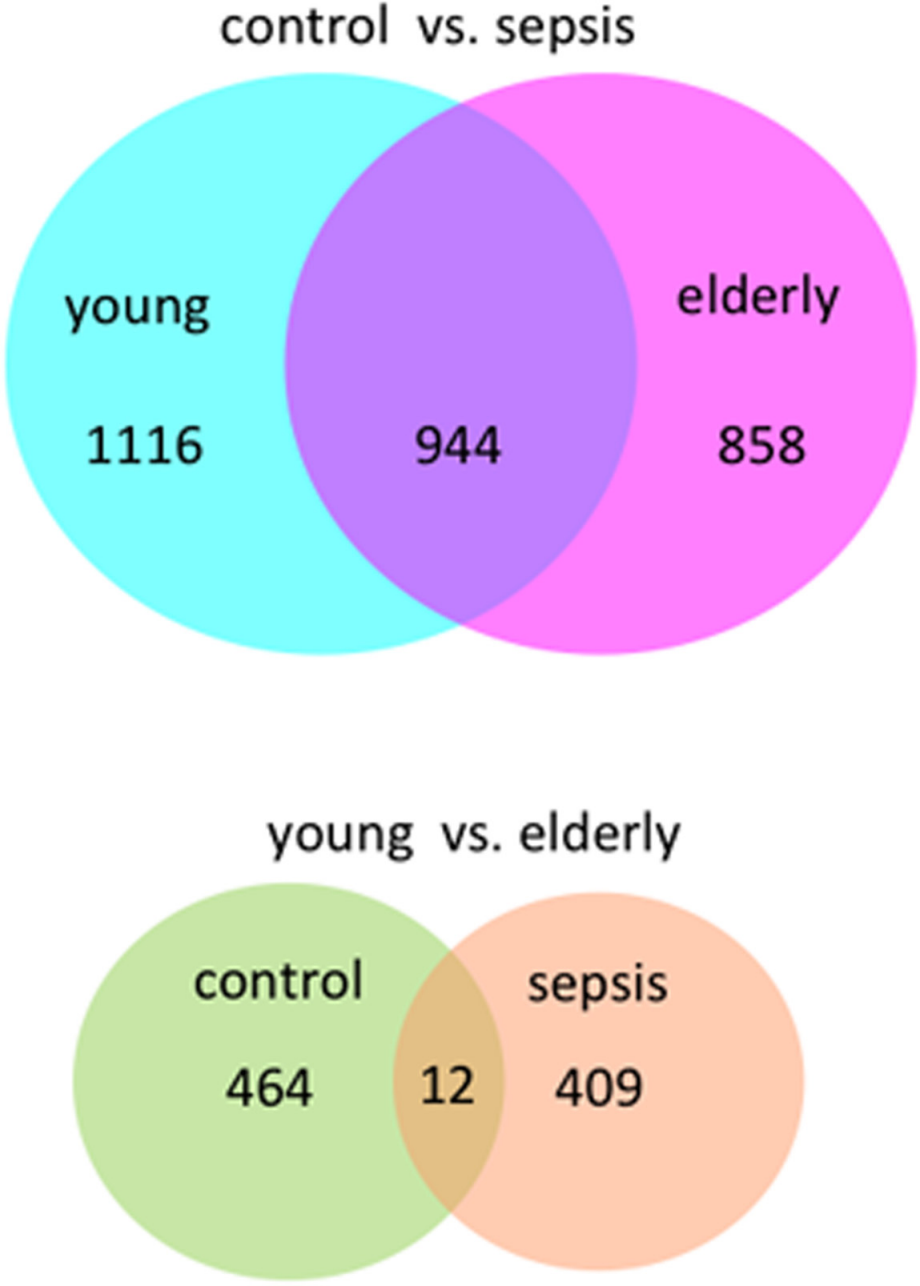
Venn diagram of differentially expressed genes in each group according to age and disease status.

We first compared gene expression profiles of neutrophils across the four groups of samples, grouped by septic status and age (Fig 1). S1 Table shows the clinical and laboratory characteristics of the study groups. As expected, the occurrence of sepsis affected the expression of a greater number of genes (4- to 5-fold) compared with aging (Table 1). We detected 421 genes that were differentially expressed between old and young subjects with sepsis, and 476 between old and young healthy controls (Fig 1). Only 1.4% of genes deregulated in old subjects compared with young subjects were common to both septic and healthy subjects. We also observed a number of lncRNAs that were deregulated in both sepsis and aging (Table 1).

To identify transcriptional changes that might explain the molecular basis of the clinical behavior in septic elderly patients, we examined the molecular pathways that were differentially expressed between elderly and young adults. First, we identified a number of canonical pathways that were enriched in genes deregulated in the elderly. Interestingly, most of these pathways were significantly enriched only in the sepsis group, i.e., genes were differentially expressed between elderly subjects with sepsis and younger adults with sepsis, with marginal enrichment in the healthy group (Table 2). The oxidative phosphorylation (*p* = 5.3 × 10^−13^) and mitochondrial dysfunction (*p* = 1.4 × 10^−10^) pathways were the most enriched in septic patients of advanced age when compared with the young septic group. Other pathways significantly enriched in the elderly were extracellular signal-regulated kinase 5 (ERK5) signaling (*p* = 9.4 × 10^−4^) and the NRF2-mediated oxidative stress response (*p* = 5.2 × 10^−3^) (Table 2). Table 3 lists the enzymes and transporters involved in oxidative phosphorylation that were differentially expressed in elderly septic patients.

**Table 2:** Differentially enriched canonical pathways between elderly and young individuals, with or without sepsis.

Canonical Pathway	Elderly vs. Young	
	Control	Sepsis
Oxidative phosphorylation	1.95E-001	5.28E-013
Mitochondrial dysfunction	8.98E-002	1.41E-010
ERK5 signaling	–	9.37E-004
NRF2-mediated oxidative stress response	2.58E-001	5.23E-003
Bile acid biosynthesis neutral pathway	1.50E-002	2.14E-001
dTMP de novo biosynthesis	1.00E+000	3.23E-003
GADD45 signaling	–	4.75E-003
DNA damage-induced 14-3-3σ signaling	–	4.75E-003
TWEAK signaling	1.22E-002	4.57E-001

Fisher’s Exact test *p*-values are shown. Significant values (*p* < 0.05) are shown.

TWEAK: TNF related weak inducer of apoptosis.

**Table 3:** Genes from the oxidative phosphorylation pathway differentially expressed between elderly and young individuals with sepsis.

Symbol	Gene name	Fold- change	p-value
NDUFA4	NADH dehydrogenase (ubiquinone) 1 alpha subcomplex, 4	0.69	1.90E-04
MT-CO1	Mitochondrially encoded cytochrome c oxidase I	0.74	2.80E-03
SDHC	Succinate dehydrogenase complex, subunit C	0.83	6.40E-03
ATP5G3	ATP synthase, H+ transporting, mitochondrial F0 complex, subunit C3	0.80	1.00E-03
COX5A	Cytochrome c oxidase subunit Va	0.83	1.90E-03
COX6C	Cytochrome c oxidase subunit Vic	0.79	5.50E-03
NDUFA8	NADH dehydrogenase (ubiquinone) 1 alpha subcomplex, 8	0.81	1.40E-03
ATP5F1	ATP synthase, H+ transporting, mitochondrial F0 complex, subunit B1	0.83	3.20E-03
NDUFB4	NADH dehydrogenase (ubiquinone) 1 beta subcomplex, 4	0.84	4.00E-03
ATP5H	ATP synthase, H+ transporting, mitochondrial F0 complex, subunit d	0.83	5.00E-03
NDUFB9	NADH dehydrogenase (ubiquinone) 1 beta subcomplex, 9	0.83	1.40E-03
COX7C	Cytochrome c oxidase subunit VIIc	0.78	2.50E-03
UQCRB	Ubiquinol-cytochrome c reductase binding protein	0.83	1.70E-03
COX11	COX11 homolog, cytochrome c oxidase assembly protein	0.81	7.50E-03
ATP5O	ATP synthase, H+ transporting, mitochondrial F1 complex, O subunit	0.82	2.40E-03
UQCRH	Ubiquinol-cytochrome c reductase hinge protein	0.82	3.10E-03
COX7A2	Cytochrome c oxidase subunit VIIa polypeptide 2	0.84	1.50E-03
UQCRQ	Ubiquinol-cytochrome c reductase, complex III subunit VII	0.85	3.00E-03

Next, we performed a pathway enrichment analysis of the genes differentially expressed in septic patients compared with healthy controls. Many pathways were identified, but none showed significantly different enrichment in the elderly compared with younger adults. Pathways that are modulated during sepsis such as Cdc42 signaling [37], phospholipase C signaling [38], interleukin 17 (IL-17) signaling [39], protein ubiquitination pathway [40], glucocorticoid receptor [41] and p38 MAPK signaling [42] were observed.

To favor the identification of molecular pathways preferentially affected in the elderly with sepsis, we performed a similar analysis using a subset of genes differentially expressed between elderly sepsis and healthy subjects, but not in younger adults (944 genes, S2 Fig). Canonical pathways preferentially altered in elderly individuals with sepsis are shown in Table 4. These pathways included transforming growth factor β (TGF-β) signaling, Wnt/β-catenin signaling, calcium signaling and other pathways of interest relevant to sepsis. Notably, during sepsis, the TGF-β pathway was modulated both in the elderly and in younger adults. However, we noted an excess of TFG-β signaling genes upregulated in elderly septic individuals but not in younger septic individuals, as well as differences in the repertoire of genes modulated upon sepsis (Table 5).

**Table 4:** Canonical pathways enriched with genes altered in elderly individuals with sepsis compared with elderly controls, but not altered in young adult septic patients compared with young adult controls.

Canonical Pathways	*p*-value
TGF-β signaling	5.8E-05
Factors promoting cardiogenesis in vertebrates	6.8E-05
Role of osteoblasts, osteoclasts and chondrocytes in rheumatoid arthritis	4.9E-04
Gα12/13 signaling	2.1E-03
Cardiomyocyte differentiation via BMP receptors	2.9E-03
Wnt/β-catenin signaling	4.2E-03
Cholecystokinin/Gastrin-mediated signaling	4.5E-03
Role of macrophages, fibroblasts and endothelial cells in rheumatoid arthritis	4.5E-03
Calcium signaling	4.7E-03
BMP signaling pathway	5.6E-03
NGF signaling	5.9E-03

Significant values (Fisher’s Exact test; *p* < 0.05) are shown.

NGF, nerve growth factor; TGF, transforming growth factor; BMP, bone morphogenetic protein.

**Table 5:** TFG-β signaling pathway genes exclusively differentially expressed in elderly individuals with sepsis compared with controls.

Symbol	Gene Name	Fold change
BMP7	Bone morphogenetic protein 7	1.19
CREBBP	CREB binding protein	1.15
INHBB	Inhibin beta B	1.53
SERPINE1	Serpin peptidase inhibitor clade E (nexin plasminogen activator inhibitor type 1) member 1	1.29
SMAD4	SMAD family member 4	1.10
SMAD9	SMAD family member 9	1.08
TGFB1	Transforming growth factor beta 1	1.13

Significant values (Fisher’s Exact test; *p* < 0.05) are shown.

A bioinformatics analysis based on the expression profiles of known targets identified by microarray identified several upstream regulators predicted to be either activated or inhibited in aging. Among those we focused on were putative regulators that had opposite activity in elderly septic individuals compared with healthy controls (Table 6). Interestingly, these included the upstream regulators of pathways detected in the previous analysis including ERK and TGF-β_1_, which were more active in septic elderly subjects compared with healthy elderly subjects, and when compared with matched young controls. This indicated that regulatory microRNAs might be predicted to be activated (miR-590-3p) or inhibited (miR-141-3p, miR-186-5p) in septic elders, as well as compounds that may exert an antagonic regulatory role in these patients compared with healthy controls (Table 6), suggesting novel potential therapeutic areas.

**Table 6:** Putative upstream regulators with inverted activity between elderly and young adults, with or without sepsis.

Upstream regulators	Elderly vs. Young adults			
	Control		Sepsis	
	z- score	*p*- value	z- score	*p*- value
miR-590-3p (miRNAs w/seed AAUUUUA)	−0.75	0.025	2.85	0.011
INSR	1.95	0.035	−1.63	0.000
ERK	−0.79	0.003	2.74	0.012
GnRH analog	−0.90	0.011	2.50	0.003
miR-141-3p (miRNAs w/seed AACACUG)	2.30	0.275	−0.95	0.020
Phorbol myristate acetate	−1.67	0.022	1.48	0.006
TP63	−0.44	0.028	1.97	0.080
Phorbol esters	−0.49	0.005	1.72	0.002
Cg (Choriogonadotropin)	−0.93	0.046	0.80	0.032
SB203580 (kinase inhibitor)	1.40	0.017	−0.16	0.012
TGFB1	−0.44	0.030	1.00	0.034
PD98059 (kinase inhibitor)	0.41	0.014	−0.55	0.019
MAP2K4	−0.76	0.007	0.15	0.015
miR-186-5p (miRNAs w/seed AAAGAAU)	0.03	0.016	−0.36	0.008

Positive/negative z-score values indicate the upstream regulator is activated/inhibited in elderly relative to young adults, respectively.

To provide independent support of the DNA microarray data analysis, quantitative PCR experiments were performed for the following genes: 1) NDUFA4 and SDHC, to investigate respiratory chain activity and 2) INHBB, TGFB1 and CREBBP, to investigate TGF-β signaling. HDAC4 (calcium signaling) and SRC (Wnt/β-catenin signaling) were also investigated because these genes were significantly altered in elderly individuals with sepsis compared with healthy elderly controls, and were not expressed at all in young adults (Table 4). In general, we observed a good association (Pearson correlation = 0.68, *p* < 0.05) between qPCR and DNA microarray measurements (S3 Fig). NDUFA4, SDHC, and INHBB were significantly altered (*p* < 0.02). Although not statistically significant, TGFB1 and HDAC4 showed transcriptional changes in agreement with those observed in the microarray. Only CREBBP and SRC showed changes that were the reverse of those observed in the microarray, but these were not statistically significant, and therefore were inconclusive. Overall, these results support our observation that aged people had a reduced expression of genes related to respiratory chain activity and an increased activation of TGF-β-related genes during the course of overwhelming infection.

## Discussion

Transcriptomics is a powerful technique used to identify therapeutic targets and biomarker signatures during infectious disease [43, 44]. A number of gene expression profiling studies were previously performed in sepsis, revealing a persistent repression of genes involved in adaptive immunity and the massive activation of innate immune pathways during septic shock [45]. Using whole blood-derived RNA, Wong et al. observed the upregulation of oxidative phosphorylation, IL-10 signaling, TLR signaling, NRF2-mediated oxidative stress responses, ubiquinone biosynthesis, TREM signaling, NF-κB signaling, protein ubiquitination pathways and IL-6 signaling in macrophages. They also observed the downregulation of T lymphocyte pathways and CCR5 signaling [46]. Similarly, Cvijanovich et al. detected the upregulation of TLR, IL-10, IL-6 and NF-κB signaling and the downregulation of T lymphocyte activation. Moreover, they detected the upregulation of acute phase responses, p38 MAPK, the complement system, and some nuclear receptor signaling molecules (LXR and PPAR) associated with the downregulation of antigen presentation pathways [47].

Using whole blood-derived RNA from patients in septic shock, the global gene expression experiments of Shanley et al. agreed with these studies. However, they also observed the upregulation of integrin, IGF-1, GM-CSF and insulin receptor signaling [48]. Similarly, Tang et al. observed the novel activation of many apoptotic genes, including *CARD12*, *APAF1* and *ELMOD2* in mononuclear cells from septic patients [49]. Surprisingly, a recent study in severe blunt trauma patients reported similar findings, with the activation of a large number of genes involved in inflammation, pattern recognition and antimicrobial functions with the simultaneous suppression of genes involved in antigen presentation and T cell proliferation, suggesting that severe physiological stress, regardless of its origin, may present common genomic signatures [50].

Sepsis affects very different groups of patients (including aged people, diabetics, patients with end-stage renal disease, trauma victims, surgical patients’, and obese people). Historically, it has been accepted that each specific subset of septic patients may have its own characteristic inflammatory immune response. In the course of sepsis, a transition from inflammation to immunosuppression has been suggested to explain the disappointing results obtained by clinical trials that investigated the use of anti-inflammatory drugs in this population [51–53]. High-throughput transcriptome analysis has provided additional information and clustering algorithms using separate training and validation cohorts suggested the existence of gene expression subtypes in sepsis and septic shock [54, 55]. Furthermore, a study of gene expression in patients with sepsis secondary to community-acquired pneumonia distinguish survivors from nonsurvivors in part by variations in the expression of genes implicated in energy metabolism [56]. In contrast, a recent systematic review based on DNA microarray expression data was unable to detect a distinctive pro-inflammatory or anti-inflammatory phase in early or late sepsis or differences in the expression patterns between groups [57]. Thus, this subject remains highly controversial.

Most transcriptome studies in sepsis were performed in a pediatric population, and to our knowledge, our study is the first genome-wide expression analysis comparing septic shock patients of advanced age with young adults.

Initially, our results confirmed that both the young and the elderly responded alike regarding the production of tumor necrosis factor α (TNFα), IL-6, IL-1β, TLRs and other classical markers of cell activation, following severe infectious stress. Some specific pathways, however, appeared to be more critically affected in the elderly with sepsis, explaining the worse prognosis observed in these patients. Based on the data shown here, it is our opinion that defects in mitochondrial function/oxidative phosphorylation, TGF-β, Wnt/β-catenin, nerve growth factor (NGF) and calcium signaling may play a major role in the clinical features of sepsis in elderly patients.

We observed a marked decrease in the expression of genes encoding components of the mitochondrial respiratory chain in the septic elderly. Impairment of mitochondrial function significantly contributed to organ failure in septic patients [58]. Physiologically, small amounts of ROS are produced at complexes I and III of the respiratory chain. Sepsis is accompanied by increased oxidative stress caused by numerous factors, including the production of ROS by neutrophils, increased xanthine oxidase activity, increased nitric oxide plasma levels, and decreased antioxidant serum capacity [59]. Pro-inflammatory mediators and oxidative stress impair the function of the enzyme complexes of the respiratory chain and lead to structural damage to mitochondrial lipids, proteins and DNA [60, 61], promoting multiple organ failure [62]. Mitochondrial damage and dysfunction secondary to oxidative stress are also observed in aged cells [3, 63, 64]. Indeed, mice that present with defective editing functions of mitochondrial DNA polymerase exhibit a markedly shortened lifespan and increased features of aging, including alopecia, kyphosis, decreased activity, and a loss of reproductive function [65].

TGF-β has pleiotropic effects on adaptive immunity, especially for the regulation of effector and regulatory CD4^+^ T cell responses. TGF-β is a potent suppressor of Th1 and Th2 effector cell differentiation, a regulator of Foxp3^+^ regulatory T cells, and is a critical cytokine for the induction of Th9 and Th17 cells [66–68]. TGF-β also has regulatory effects on CD8^+^ cells [69] and promotes phagocytosis [70]. Thus, we propose that increased activation of the TGF-β pathway in elderly septic patients may induce a prolonged downregulation of adaptive immunity and an increased Th17 response.

The Wnt/β-catenin pathway determines major developmental processes in the embryo and regulates maintenance, self-renewal and differentiation of adult mammalian tissue stem cells [71]. Many organs with a high cell turnover require continuous replenishment by somatic stem cells. Ageing of hematopoietic stem cells is associated with impaired hematopoiesis in the elderly [72, 73], which was attributed to a switch from canonical to non-canonical Wnt signaling [74]. In addition, a recent study reported that Wnt/β-catenin signaling induced the aging of mesenchymal stem cells through ROS generation [75]. These mechanisms might be responsible for defects in dendritic cell differentiation [76] and loss of T cell potential observed with age [77]. The Wnt/β-catenin pathway participates in the regulation of many other pathways, including NF-κB and PI3-kinase pathways, with important implications in the pathophysiology of septic shock [78]. In our opinion, the increased upregulation of the Wnt/β-catenin pathway in the elderly might be a critical detrimental factor.

Nerve growth factor (NGF) ensures the maintenance of phenotypic and functional characteristics of several populations of neurons and immune cells. The role of NGF has never been investigated in sepsis, but might be involved because neuropeptides possess a broad number of regulatory functions in immunity [79] and NGF directly affects the survival and differentiation of stem cells, granulocytes, lymphocytes and monocytes [80–83]. Further studies should investigate its role in sepsis and in specific populations of septic patients, such as the elderly.

Calcium has a myriad of cellular functions, including effects on hormone secretion, enzyme activity, nerve conduction, and muscle contraction with obvious repercussions in sepsis. Calcium influx is a potent cell activator and, together with phosphate, alters electrostatic fields and protein conformation, dictating the majority of signal transduction [84].

The mechanisms that lead to the dysfunction of specific pathways in the immune responses of older people are complex and might involve multiple factors. We propose that environmental factors [85, 86], microRNAs [87] and epigenetic changes [88] might play a key role as modulating factors of the immune pathways that are particularly affected in older people.

Our data confirm previous reports that aging is accompanied by changes in the expression of genes related to immune responses [89], and further studies are required to support the involvement of pathways we identified and their relevance in sepsis. Interestingly, our analysis also suggests that a number of upstream regulators of molecular pathways display an inverted pattern of expression in elderly patients with sepsis compared with healthy controls, including microRNAs miR-590-3p (predicted activation in the septic elderly), miR-141-3p (predicted inhibition in the septic elderly) and miR-186-5p (predicted inhibition in the septic elderly). Another distinctive feature of this study was the observation for the first time that subsets of long noncoding RNAs are deregulated in the immune system in sepsis and aging, warranting additional studies aimed at investigating the biological roles exerted by this class of transcript in septic shock in patients of advanced age.

## Conclusions

There is great hope that high-throughput screening technology will result in a better understanding of the complexity of systemic inflammation, identifying new therapeutic targets and groups of patients that may benefit from specific interventions.

Here, we identified major pathways preferentially deregulated in the elderly following severe infection, providing evidence that the systemic inflammatory response differs according to patient age. Thus, oxidative stress might have a major role in inducing multiple dysfunctions in the elderly. Furthermore, our study identified additional important genes and molecular pathways, thus helping to elucidate the mechanisms involved in signaling crosstalk during infection and in aging.

## Supporting Information

S1 Fig Hierarchical clustering of samples from sepsis (S) and healthy (H) subjects based on gene expression measurements. After background filtering (see “Methods” for details), valid measurements from 16,698 genes were used to group samples using UPGMA hierarchical clustering and Pearson correlation as a distance measurement. Dendrograms are colored according to disease status (sepsis in red, healthy controls in blue) and age (elderly subjects are shown in darker colors whereas those from young adults are shown in lighter colors). Samples are well grouped by the disease status sepsis/healthy and moderately grouped by age.
(DOCX)

S2 Fig Venn diagram showing all possible intersections in differentially expressed genes between all groups. (DOCX)

S3 Fig Confirmation of gene expression changes in sepsis and age detected by microarray using Real-Time PCR. Genes with comparable changes in gene expression are shown in green and with an inverted pattern in red. Statistically significant results are shown in dark green. Values that follow a trend, but do not reach statistical significance are shown in light green.
(DOCX)

S1 Table Patient characteristics. (DOCX)
