# Supplementary material for: Septic Shock in Advanced Age: Transcriptome Analysis Reveals Altered Molecular Signatures in Neutrophil Granulocytes
Source: PLoS One. 2015 Jun 5;10(6):e0128341. doi: 10.1371/journal.pone.0128341 (PMC4457834; doi:10.1371/journal.pone.0128341)
Supplement: S1 Fig — After background filtering (see “Methods” for details), valid measurements from 16,698 genes were used to group samples using UPGMA hierarchical clustering and Pearson correlation as a distance measurement. Dendrograms are colored according to disease status (sepsis in red, healthy controls in blue) and age (elderly subjects are shown in darker colors whereas those from young adults are shown in lighter colors). Samples are well grouped by the disease status sepsis/healthy and moderately grouped by age. (DOCX) [file pone.0128341.s001.docx]

**S1 Fig. Hierarchical clustering of samples from sepsis (S) and healthy (H) subjects based on gene expression measurements.**


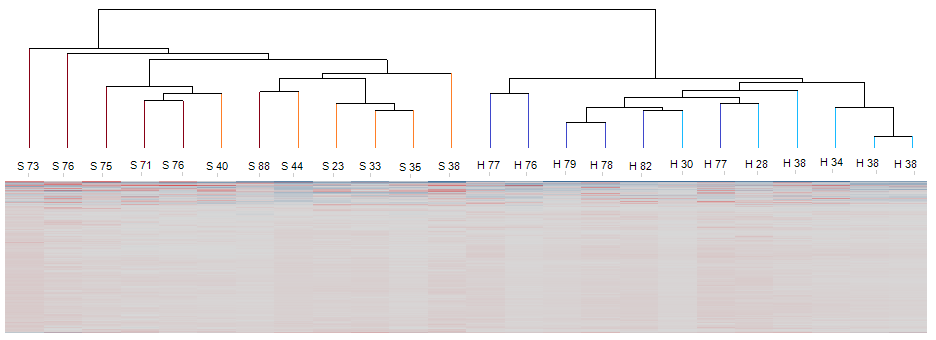


After background filtering (see “Methods” for details), valid measurements from 16,698 genes were used to group samples using UPGMA hierarchical clustering and Pearson correlation as distance measurement. Dendrograms are colored according to disease status (sepsis in red, healthy controls in blue) and age (elderly subjects are shown in darker colors whereas those from young adults are depicted in lighter colors). It is apparent that the samples are well grouped by the disease status sepsis/healthy and moderately grouped by age.
