# Supplementary material for: Septic Shock in Advanced Age: Transcriptome Analysis Reveals Altered Molecular Signatures in Neutrophil Granulocytes
Source: PLoS One. 2015 Jun 5;10(6):e0128341. doi: 10.1371/journal.pone.0128341 (PMC4457834; doi:10.1371/journal.pone.0128341)

**S2 Fig. Venn diagram showing all possible intersections in differentially expressed genes between all groups.**


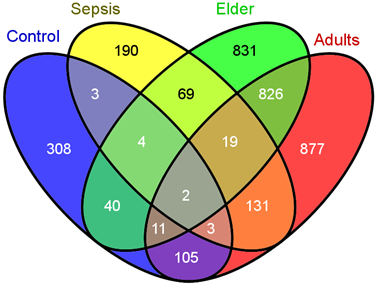

Supplement: S2 Fig — (DOCX) [file pone.0128341.s002.docx]
