# Supplementary material for: Septic Shock in Advanced Age: Transcriptome Analysis Reveals Altered Molecular Signatures in Neutrophil Granulocytes
Source: PLoS One. 2015 Jun 5;10(6):e0128341. doi: 10.1371/journal.pone.0128341 (PMC4457834; doi:10.1371/journal.pone.0128341)
Supplement: S3 Fig — Genes with comparable changes in gene expression are shown in green and with an inverted pattern in red. Statistically significant results are shown in dark green. Values that follow a trend, but do not reach statistical significance are shown in light green. (DOCX) [file pone.0128341.s003.docx]

**S3 Fig. Confirmation of gene expression changes in sepsis and age detected by microarray using Real-Time PCR.**


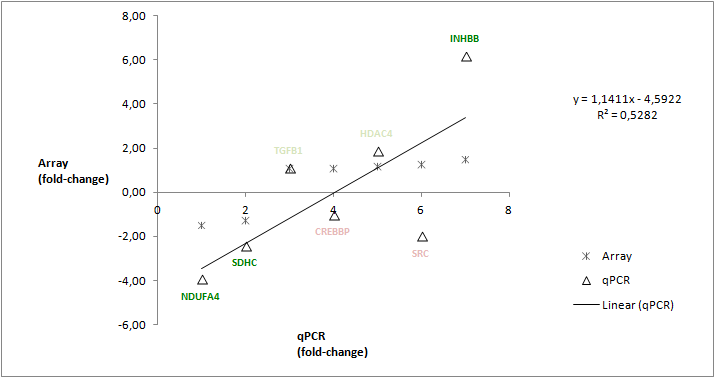


Genes with comparable changes in gene expression are shown in green and with an inverted pattern in red. Statistically significant results are shown in dark green. Values that follow a trend, but do not reach statistical significance are shown in light green.
