# Supplementary material for: Septic Shock in Advanced Age: Transcriptome Analysis Reveals Altered Molecular Signatures in Neutrophil Granulocytes
Source: PLoS One. 2015 Jun 5;10(6):e0128341. doi: 10.1371/journal.pone.0128341 (PMC4457834; doi:10.1371/journal.pone.0128341)
Supplement: S1 Table — (DOCX) [file pone.0128341.s004.docx]

**S1 Table. Patient characteristics.**

| Sample characteristics |  | Healthy controls | | Sepsis | |  |
| --- | --- | --- | --- | --- | --- | --- |
| Variable (mean ± SD) | All | Young adults | Elderly | Young adults | Elderly | Adult vs. Elderly (*p-*value) |
| Age (years) | 57 ± 22 | 34 ± 5 | 78.1 ± 2.1 | 36 ± 7 | 76 ± 6 | < 0.0001 |
| Male sex | 22 (92%) | 5 (83%) | 6 (100%) | 5 (83%) | 6 (100%) |  |
| Clinical Variables: |  | Expected values for healthy subjects | |  |  |  |
| Urea (mg/dl) | 79 ± 41 | 10–50 | | 64 ± 36 | 95 ± 43 | 0.22 |
| Creatinine (mg/dl) | 1.6 ± 0.8 | 0.70–1.20 | | 1.5 ± 1.0 | 1.7 ± 0.6 | 0.69 |
| Sodium (mEq/L) | 142 ± 9 | 135–145 | | 146 ± 8 | 139 ± 9 | 0.19 |
| Potassium (mEq/L) | 4.1 ± 1.0 | 3.5–5.0 | | 4.0 ± 1.1 | 4.2 ± 1.1 | 0.76 |
| Magnesium (mg/dl) | 1.9 ± 0.4 | 1.58–2.55 | | 1.9 ± 0.5 | 1.9 ± 0.4 | > 0.99 |
| Ionized calcium (mg/dl) | 4.7 ± 1.0 | 4.60–5.30 | | 5.0 ± 1.4 | 4.4 ± 0.4 | 0.36 |
| Hb (g/dl) | 11.5 ± 2.5 | 13.0–18.0 | | 10.1 ± 1.9 | 13.0 ± 2.3 | 0.04 |
| Leucocytes (1000/mm3) | 13 ± 7 | 4–11 | | 12 ± 5 | 14 ± 10 | 0.67 |
| Polymorphonuclear cells (1000/mm3) | 11 ± 7 | 1.8–7 | | 10 ± 5 | 12 ± 9 | 0.65 |
| Platelets (1000/mm3) | 177 ± 97 | 150–350 | | 167 ± 115 | 188 ± 86 | 0.73 |
| C-reactive protein (mg/dl) | 251 ± 141 | < 0.5 | | 290 ± 146 | 205 ± 134 | 0.32 |
| ICU case-fatality |  |  | | 2 (33%) | 3 (50%) |  |
| Source of infection |  |  | | pneumonia (n = 2),  pulmonary abscess (n = 1),  urinary infection (n = 3) | bacteremia (n = 2),  pneumonia (n = 2),  urinary infection (n = 2) |  |
| Type of infection |  |  | | unknown (n = 1),  *Klebsiella pneumoniae* (n = 1),  *Haemophilus influenzae* (n = 1),  *Escherichia coli* (n = 1),  *Acinetobacter baumannii* (n = 2) | *Streptococcus agalactiae* (n = 1),  unknown (n = 4),  *Staphylococcus aureus* (n=1) |  |

ICU, intensive care unit; Hb, hemoglobin.
